# Escape and absconding among offenders with schizophrenia spectrum disorder – an explorative analysis of characteristics

**DOI:** 10.1186/s12888-021-03117-1

**Published:** 2021-03-04

**Authors:** Johannes Kirchebner, Steffen Lau, Martina Sonnweber

**Affiliations:** Department of Forensic Psychiatry, University Hospital of Psychiatry Zurich, Zurich, Switzerland

**Keywords:** Absconding, Escape, Schizophrenia, Offending, Machine learning, Forensic psychiatry

## Abstract

**Background:**

Escape and absconding, especially in forensic settings, can have serious consequences for patients, staff and institutions. Several characteristics of affected patients could be identified so far, albeit based on heterogeneous patient populations, a limited number of possible factors and basal statistical analyses. The aim of this study was to determine the most important characteristics among a large number of possible variables and to describe the best statistical model using machine learning in a homogeneous group of offender patients with schizophrenia spectrum disorder.

**Methods:**

A database of 370 offender patients suffering from schizophrenia spectrum disorder and 507 possible predictor variables was explored by machine learning. To counteract overfitting, the database was divided into training and validation set and a nested validation procedure was used on the training set. The best model was tested on the validation set and the most important variables were extracted.

**Results:**

The final model resulted in a balanced accuracy of 71.1% (95% CI = [58.5, 83.1]) and an AUC of 0.75 (95% CI = [0.63, 0.87]). The variables identified as relevant and related to absconding/ escape listed from most important to least important were: more frequent forbidden intake of drugs during current hospitalization, more index offences, higher neuroleptic medication, more frequent rule breaking behavior during current hospitalization, higher PANSS Score at discharge, lower age at admission, more frequent dissocial behavior during current hospitalization, shorter time spent in current hospitalization and higher PANSS Score at admission.

**Conclusions:**

For the first time a detailed statistical model could be built for this topic. The results indicate the presence of a particularly problematic subgroup within the group of offenders with schizophrenic spectrum disorder who also tend to escape or abscond. Early identification and tailored treatment of these patients could be of clinical benefit.

**Supplementary Information:**

The online version contains supplementary material available at 10.1186/s12888-021-03117-1.

## Background

In the mental health system, escape is defined as gaining freedom by breaking through the secured perimeter, in forensic psychiatric hospitals often the outer wall or fence of a ward [1, 2], while absconding is defined as gaining freedom by evading the supervision of staff during a controlled opening outside the ward or hospital [1]. As forensic psychiatry is defined as “a subspecialty of psychiatry in which scientific and clinical expertise is applied in legal contexts involving civil, criminal, correctional, regulatory, or legislative matters, and in specialized clinical consultations in areas such as risk assessment or employment” this is a crucial area of research and daily clinical practice in this field.

Both escape and absconding from closed psychiatric wards, and especially from secure forensic hospitals, can be serious events leading to potentially grave outcomes [1–5]. These can directly affect the patient concerned, such as overdosing [6–8], self-inflicted injury [8–10], attempted suicide [10–14] or completed suicide [15, 16], prolonged rehabilitation and treatment [17], medication non-compliance [17], violence against others [4, 9, 14] or (re-) offending [8, 13, 15, 18–22].

The reputation of institution and staff can also be damaged by such incidents and subsequent negative media coverage regarding mental health and security may lead to increased stigma, reduced confidence in the particular institution or the health care system as a whole, anger and guilt among staff, stress and anxiety among patients [4, 23–25].

Despite those detrimental effects, research on this topic is rather sparse [1, 2]. Recent reviews have identified 39 studies on escape and absconding in general psychiatry [2, 17], but only nine on the subject in forensic psychiatry, although the consequences are particularly severe in the latter setting [23].

Most of the relevant research on escape and absconding was conducted by Bowers [9, 26–28], who focused primarily on the analysis of the characteristics of affected patients. Subsequent studies on this topic were based on these characteristics and were predominantly undertaken in the United Kingdom and Australia. The diagnosis of schizophrenia spectrum disorder (SSD) has been identified as one of the most influential characteristics for escape or absconding with incidences of up to 71% in patients examined [1, 4, 5, 17, 23, 24, 27–33]. Further risk factors for escape or absconding included: younger age [1, 3–5, 9, 29, 33–36], male sex [1, 4, 5, 29, 30, 33, 36–38], being unemployed [4, 37, 39], being unmarried [1, 5, 7, 10, 36, 39], homelessness [38], the number of diagnoses [38], having a personality disorder [1, 4, 5, 11] or affective disorder [4, 5, 9, 33, 36], prior convictions [4, 17, 19], forensic psychiatric treatment in the past [1], absconding in the past [1, 5, 23, 24, 30, 32, 35, 39], compulsory detainment [1, 5, 38], treatment non-compliance [1] and medication non-compliance in the past [5], exacerbation of symptoms [19, 28, 31], aggressive behavior [24, 38], alcohol or drug abuse [1, 4, 5, 23, 24, 33, 38–40], a history of sexual abuse [1], not having a history of self-inflicted harm [38], suicidal behavior [23], shorter duration of current hospitalization [3, 4, 33, 34, 38, 41] and also longer duration of current hospitalization [23, 30].

Although past research has provided important insights into the topic, certain weaknesses are apparent: (1) Previous studies included heterogeneous patient populations with different diagnoses. Although individuals diagnosed with SSD constitute the majority of escapees and absconders, there is no study to date that has examined these patients exclusively (2). The investigated features are the result of the valuable work of Bowers and his colleagues and were reproduced in subsequent studies. However, new and potentially more specific factors could not be uncovered so far. (3) The statistical analyses of earlier studies were mostly based on null hypothesis significance testing (NHST), in rare cases on linear regression methods, which was already recognized as a problem in 2000 [42], yet received only limited attention in further studies. The problem with examining multiple single variables with NHST, known as multiple testing [43, 44], is that each statistical test is associated with a false positive rate (type I error) and continues to accumulate with each additional test. This results in variables being falsely identified as significant and is further exacerbated when there is no correction for multiple testing [45, 46]. Another limitation of the NHST is the insufficient consideration of interactions between the variables and that no evaluations of the quality of statistical models are provided. Simply looking at the significant variables may produce models and interpretations of limited informative value. Therefore, the aim of this study is to investigate the phenomenon of escape and absconding employing a complex data set of 370 offenders with SSD and over 500 potential influential variables via machine learning.

### Machine learning

Due to technical and scientific progress in the fields of mathematics and computer science, it is now possible to perform statistical calculations and pattern recognition using complex statistical algorithms. In this context, the term machine learning (ML) is commonly used. The advantages of ML are manifold: large amounts of data can be processed quickly, a multitude of possible variables and their influence on each other can be investigated, complex nonlinear relationships can be calculated, and predictive models with different performance measures can be built and evaluated. This also appears to be a promising approach for new analyses in the medical sciences. In psychiatry, ML is already used in several areas, such as neuroimaging and clinical decision making. In the subfield of forensic psychiatry, its application is still relatively rare. However, in this area, where basic knowledge is somewhat scarce but extensive datasets may already exist, ML represents a promising opportunity to gain new insights - for example regarding the characteristics leading to escape or absconding (for more information on ML see [47–50]).

### Objectives

Using ML algorithms, the aim of this exploratory study was to identify the most influential parameters in an extensive database distinguishing between patients who escape or abscond and those who do not, based on the unique group of forensic offender patients with SSD, and to quantify the performance of the calculated model.

## Methods

### Setting

Data was obtained in a single mental health facility, Switzerland’s largest forensic psychiatric clinic specialized in the treatment of patients suffering from schizophrenia or other acute psychiatric pathology. The clinic holds a total of 79 beds and offers court mandated treatment (often for several years) for patients who have committed a crime or regular prisoners whose mental health status does not allow treatment within prison. Patients are treated in different levels of care and security according to treatment effort and reduced dangerousness. According to “The Matrix of Security” [51], 27 of the beds are within a high-security setting where no leaves are permitted. 39 beds are in closed wards with medium- to low-security settings, and 13 are in an open ward with low-security setting. Leaves are approved after a detailed assessment process (often reviewed by judicial authorities) that begins with short walks accompanied by professional staff in one-on-one supervision. If treatment leads to an improvement in psychopathology and reduces the risk of future offending, the patient may be allowed to leave the ward unaccompanied, initially for a short time, and later for a longer period, to expedite rehabilitation.

### Source of data and measures

The files of 370 offender patients diagnosed with SSD as defined in ICD-9 [52] or ICD-10 [53] were analyzed retrospectively. The coding protocol covered the following domains: social-demographic data, childhood/ youth experiences, psychiatric history, past criminal history, social and sexual functioning, details on the offence leading to forensic hospitalization, prison data, particularities of the current hospitalization and psychopathological symptoms by closely adopting the positive and negative symptom scale (PANSS), whereby symptoms were divided into the usual 30 sub-categories and rated on a scale (completely absent, discretely present or substantially present). Our extensive database has already been used in other studies and is part of a larger project in which the medical records of forensic inpatients have been analyzed in detail to obtain insights into the under-researched area of SSD and criminal behavior. Full details on data collection and processing can be found in Kirchebner et al. [54] and Kirchebner et al. [55].

### Statistical procedures – machine learning

**Fig. 1:**
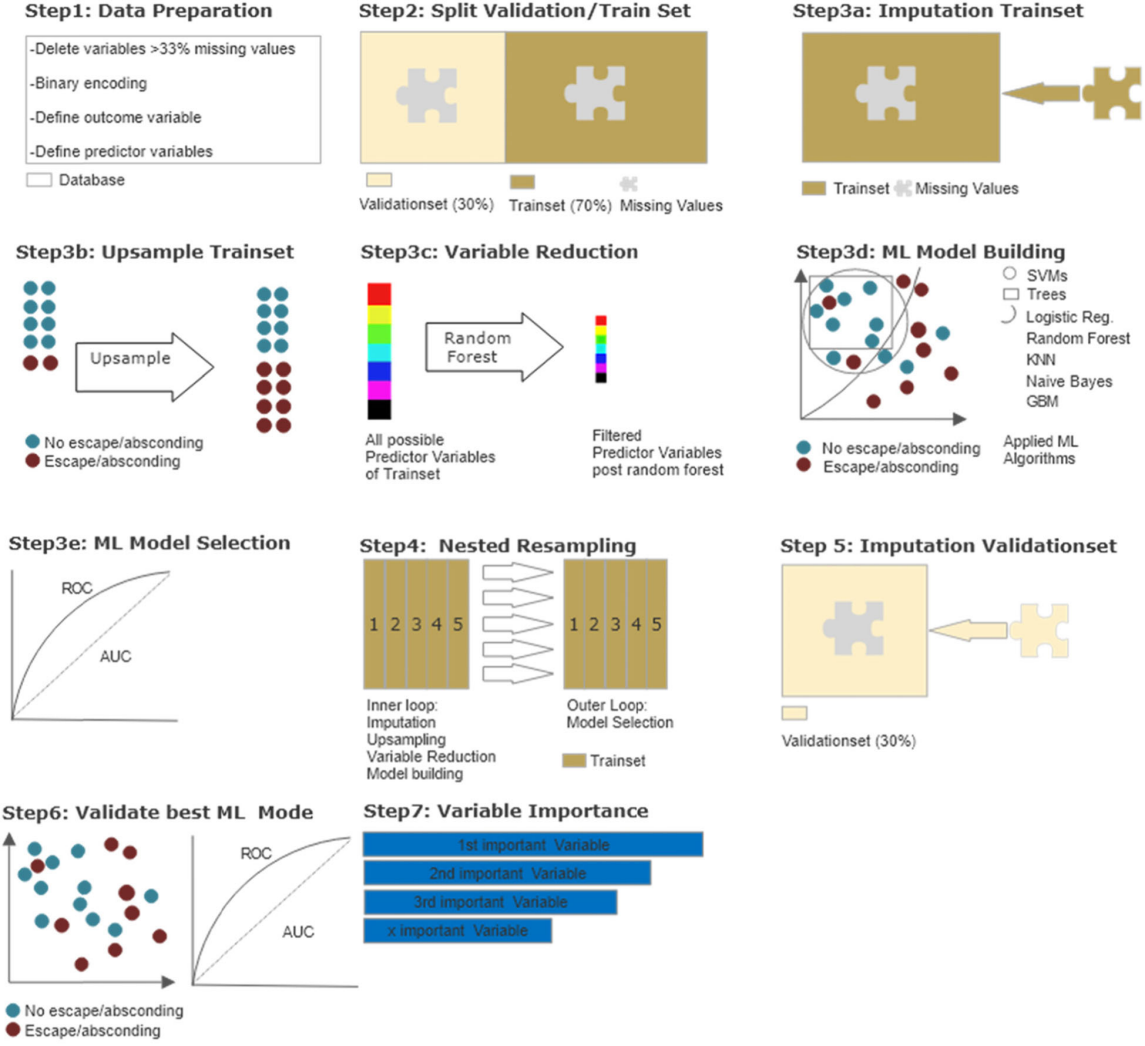
Overview of statistical procedures. *Step 1 – Data Preparation*: Multiple categorical variables were converted to binary code. Continuous and ordinal variables were not manipulated. Outcome variable escape or absconding vs. no escape or absconding and 507 predictor variables were defined. *Step 2 – Datasplitting*: Split into 70% training dataset and 30% validation dataset*. Step 3 a, b, c, d, e – Model building and testing on training data I*: Imputation by mean/mode; upsampling of outcome “escape/absconding” ×7; variable reduction via random forest; model building via ML algorithms - logistic regression, trees, random forest, gradient boosting, KNN (k-nearest neighbor), support vector machines (SVM), and naive bayes; testing (selection) of best ML algorithm via ROC parameters. *Step 4 – Model building and testing on training data II*: Nested resampling with imputation, upsampling, variable reduction and model building in inner loop and model testing on outer loop. *Step 5–5 Model building and testing on validation data I*: Imputation with stored weights from Step 3a. *Step6 – Model building and testing on validation data II:* Best model identified in Step 3e applied on imputed validation dataset and evaluated via ROC parameters. *Step7:* Sensitivity analysis and ranking of variables by indicative power

Parts of the following section were published in Günther et al. [51] and are partially adopted here and extended to include the methodology of the current research question. Given the explorative nature of this study, supervised machine learning (ML) appeared to be the optimal method to identify the most important influencing factors of a variety of variables and to determine the model providing the best predictive power. An Overview of the statistical steps can be seen in Fig. 1 and are further described below. All Steps were performed using R version 3.6.3. and the MLR package v2.171 [56]. CI calculations of the balanced accuracy were conducted using MATLAB R2019a (MATLAB and Statistics Toolbox Release 2012b, The MathWorks, Inc., Natick, Massachusetts, United States) with the add-on “computing the posterior balanced accuracy” v1.0 [57].

#### Preliminary data processing and measures

All raw data was first prepared for machine learning (see Fig. 1 Step 1) - multiple categorical variables were transformed to binary code. Continuous and ordinal variables were not adjusted. Owing to the retrospective nature of the study and the large number of variables collected, there were missing values, especially for information on patients’ in-depth biographical history. Variables with more than 33% missing values were eliminated resulting in a remaining set of 508 variables.

The outcome variable – patient, who escaped or absconded – was dichotomized into (1) patient escaped/ absconded and (2) patient did not escape/ abscond. The following incidents were considered as an event of absconding: running away from a member of staff or refusing to return to the unit with a member of staff, whilst on escorted leave or failure to return from unescorted leave. Escape was defined as successful or unsuccessful attempt to flee from the perimeter of the hospital.

Patients who were hospitalized in the high security area of our forensic hospital were excluded from the study because no (accompanied) leaves are permitted in this phase of treatment and no escape has ever occurred from this area. 274 patients remained for further analysis. Of these patients, 34 (12.4%) were involved in an event of escape or absconding and 240 (87.6%) were not. Non-escape/ absconding was defined as the positive class, escape/ absconding as the negative class.

The initial dataset was randomly divided into two subsets (see Fig. 1, Step 2) - a training dataset with 70% of all patient cases (191 patients) and a validation dataset with 30% of cases (83 patients). The training data set was utilized for variable reduction and model building/ selection (see Fig. 1, Steps 3x and 4) while the validation data set was applied to evaluate the prior selected statistical model (see Fig. 1, Step 5, 6 and 7). Predictor variable selection, model building and model evaluation were based on different subsets of the existing data to reduce the risk for overfitting.

#### Imputation, balancing, variable filtering, statistical model building/ selection and nested resampling

All the subsequent steps under above section were conducted with the training data set (191 patients) only, while the data set for validation (83 patients) remained unchanged:

In order to flexibly apply all ML algorithms, imputation of missing values was performed. Imputation by mean for continuous variables and by mode for categorical variables included in the MLR package was applied (see Fig. 1, step 3a). The imputation weights were saved for later reuse on the validation dataset (see Fig. 1, step 5).

With a ratio of 12.4 to 87.6%, the outcome variable was unevenly distributed. To create the most balanced result possible, random up sampling at a rate of 7 was used, leading to a more balanced result of 50.2 to 49.8% (see Fig. 1, Step 3b).

A main objective of the present study was to identify the most important predictor variables out of 507 possible variables. In addition, a reduction of variables can counteract overfitting and keep computation times at an acceptable level during initial model building. To this end, variable reduction to the 10 most important predictors was performed using the randomForestSRC package implemented in the MLR package, which evaluates variable importance [58] (see Fig. 1, step 3c).

Since our database of 274 observations is relatively small for machine learning purposes and we focused on variable extraction and prediction, we applied discriminative model building with logistic regression, trees, random forest, gradient boosting, KNN (k-nearest neighbor), support vector machines (SVM) and as an easily applicable generative model building, naive Bayes (for a more detailed description see [59]) (Fig. 1, Step 3d). No hyperparameters were optimized. The default hyperparameters can be obtained from the supplementary material.

The model performance of each model was calculated and assessed in terms of its balanced accuracy (the average of true positive and true negative rate, which is better suited for model evaluation and calculation of confidence intervals in imbalanced data [57]) and goodness of fit (measured with the receiver operating characteristic, balanced curve area under the curve method, ROC balanced AUC) [60]. Moreover, specificity, sensitivity, positive predictive value (PPV) and negative predictive value (NPV) were evaluated. As our training dataset was artificially balanced, the model with the highest AUC was chosen for final model validation on the validation dataset [60] (see Fig. 1, Step 3e).

Finally, the set of identified variables was tested for multicollinearity to avoid dependencies between the variables.

To avoid overfitting, it is advisable to include imputation, filtering, balancing and model building in a cross validation process kept separate from model-testing [61, 62].

Nested resampling seems best suited for this objective – in an inner loop data processing and model training are performed imbedded in cross-validation and then in an outer loop the performance of these models is tested also embedded in cross-validation. In this study the nested resampling model was built with the inner loop performing imputation, oversampling, variable filtering and model building within 5-fold cross-validation and the outer loop being used for performance evaluation also embedded in 5-fold-cross-validation, a technique of artificially creating different subsamples of a data set [63] (see Fig. 1, Step 4).

#### Model validation and variable importance

The validation subset of the total data (30%, 83 patients) was imputed with the stored weights from Step 3a by mean and mode (see Fig. 1, Step 5a). Then, the best previously identified model was applied to the data and again the performance measures of this final model were assessed (see Fig. 1, Step 6). The variables used to predict the outcome variable (patient escaped/ absconded and patient did not escape/ abscond) in the final model were sorted by indicative power through means of a sensitivity analysis using the rminer package [64] (see Fig. 1, Step 7).

## Results

An overview of the performance parameters of the different calculated models during the nested resampling procedure can be found in Table 1. With a balanced accuracy of 76.7%, (95% CI = [68.4, 82.7]) and an AUC of 0.88 (95% CI = [0.81, 0.95]) naïve bayes outperformed all other ML algorithms.

**Table 1:** Machine learning models and performance in nested cross-validation on training dataset – escape/ absconding vs. no escape/ absconding

Statistical Procedure	Balanced Acc. (%) (95% CI)	AUC (%) (95% CI)	Sensitivity (%) (95% CI)	Specificity (%) (95% CI)	PPV (%) (95% CI)	NPV (%) (95% CI)
Logistic Regression	73.9 [64.7, 79.8]	0.81 [0.72, 0.89]	80.2 [76.0, 84.4]	67.6 [52.5, 82.7]	95.8 [93.5, 98.1]	26.9 [17.9, 35.9]
Tree	72.6 [64.2, 79.6]	0.80 [0.71, 0.89]	81.9 [64.5, 92.3]	63.2 [47.8, 78.5]	95.3 [92.8, 97.7]	27.9 [18.4, 37.4]
Random Forest	71.1 [61, 76.6]	0.84 [0.76, 0.92]	94.8 [92.4, 97.1]	47.4 [31.5, 63.2]	94.2 [91.8, 96.7]	50.0 [33.7, 66.3]
Gradient Boosting	65.8 [58.4, 73.8]	0.82 [0.74, 0.90]	89.5 [86.3, 92.8]	42.1 [26.4, 57.8]	93.3 [90.6, 96.0]	30.8 [18.2, 43.3]
KNN	66.5 [58.6, 74.3]	0.85 [0.59, 0.99]	85.5 [81.7, 89.1]	47.4 [31.5, 63.2]	93.6 [90.9, 96.3]	26.5 [15.9, 36.9]
SVM	73.4 [65.5, 80.9]	0.84 [0.76, 0.92]	94.2 [91.7, 96.7]	52.6 [36.8, 68.5]	94.7 [92.3, 97.1]	50.0 [34.5, 65.5]
Naive Bayes	76.7 [68.4, 82.7]	0.88 [0.81, 0.95]	79.7 [75.4, 83.9]	73.7 [59.7, 87.7]	96.5 [94.3, 98.6]	28.6 [19.6, 37.5]

*AUC* area under the curve (level of discrimination), *PPV* positive predictive value, *NPV* negative predictive value, *KNN* k-nearest neighbors, *SVM* support vector machines

The quality of the final model in the validation step is shown in Table 2. As expected, the balanced accuracy of 71.1% (95% CI = [58.5, 83.1]) and an AUC of 0.75 (95% CI = [0.63, 0.87]) were less than the results of the initial training model, but still meaningful. With a sensitivity of 88.2% (95% CI = [87.9, 88.5]) over three-quarters of patients who did not escape or abscond were identified correctly, with a specificity of 60% (95% CI = [59.2, 60.8]), less than two-thirds of patients, who escaped or absconded were detected correctly.

**Table 2:** Final naïve bayes model performance measures on validation dataset - escape/ absconding vs. no escape/ absconding

Performance measures	%	95% Confidence Interval
Balanced Accuracy	71.1	[58.5, 83.1]
AUC	0.75	[0.63, 0.87]
Sensitivity	88.2	[87.9, 88.5]
Specificity	60.0	[59.2, 60.8]
PPV	90.9	[90.7, 91.1]
NPV	52.9	[52.2, 53.7]

The absolute and relative distribution of the 10 most influential variables among the whole dataset can be seen in Table 3. In addition to age at diagnosis, age at admission and number of index offences, the other influential variables covered circumstances related to the current hospitalization. Testing for multicollinearity showed no dependencies between the variables (detailed results see supplementary materials).

**Table 3:** Absolute and relative distribution of indicative variables on complete dataset - escape/ absconding vs. no escape/ absconding

Variable description	Escape/absconding		No escape/absconding	
	n/N (%)	mean (SD)	n/N (%)	mean (SD)
Age at admission		31.1 (6.8)		34.1 (10.2)
Age at SSD diagnosis		24.4 (5.6)		29.0 (9.6)
Dissocial behavoir reported during current hospitalisation	25/34 (73.5)		96/238 (40.3)	
Events of rule breaking during current hospitalization	22/34 (64.7)		50/236 (21.2)	
Amount of index offences		2.9 (2.2)		2.1 (1.7)
Daily cumulative olanzapine equivalent at dischargeª		24.1 (15.2)		18.6 (14)
Forbidden intake of drugs during current hospitalisation	14/34 (41.2)		11/240 (4.6)	
Time spent in current forensic hospitalization (in weeks)		140.7 (126.8)		145.7 (142.7)
PANSS Score at admission		25.4 (12.8)		24.3 (12.4)
PANSS Score at discharge		11.9 (9.5)		11.4 (10.1)

*SD* Standard deviation, *PANSS* positive and negative syndrome scale

ªTo ensure comparability between different studies, daily neuroleptic doses were converted to olanzapine equivalents by using conversion factors derived from the classical weighted average dose method [65], the minimum effective dose method [66] or, in all other cases, from olanzapine equivalents based on international expert consensus [67]

**Fig. 2:**
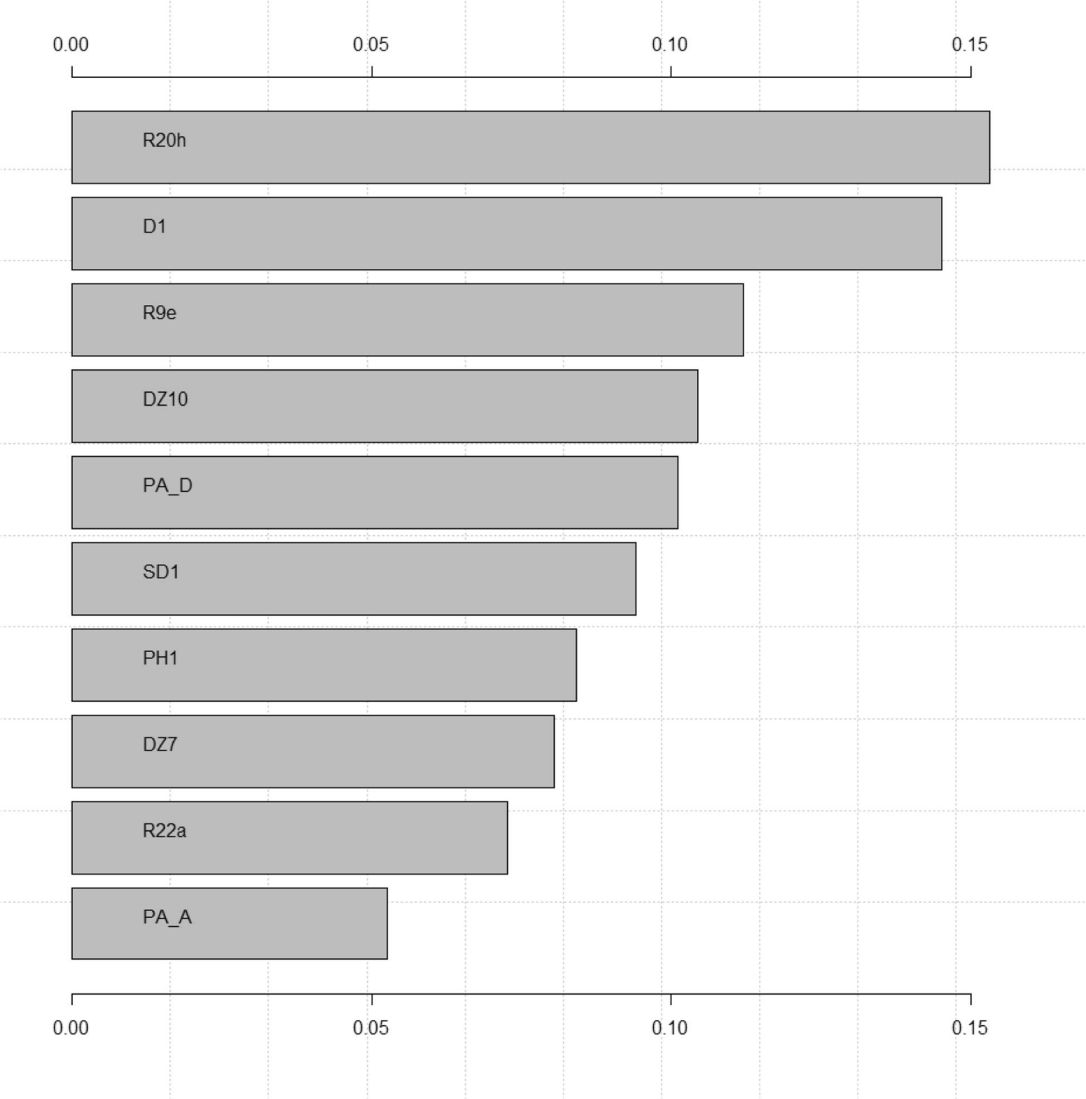
Variable importance of final model escape/ absconding vs. no escape/ absconding: R20h = Forbidden intake of drugs during current hospitalization; D1 = Amount of index offences; R9e = Daily cumulative olanzapine equivalent at discharge; DZ10 = Events of rule breaking during current hospitalization; PA_D = PANSS Score at discharge; SD1 = Age at admission; PH1 = Age at SSD diagnosis; DZ7 = Dissocial behavior reported during current hospitalisation; R22a = Time spent in current forensic hospitalization (in weeks); PA_A = PANSS Score at admission

A one-sided tornado graph comparing the relative importance of the identified variables during model validation is presented in Fig. 2. It shows the effect on the output variable (patient escaped/ absconded and patient did not escape/ abscond) by varying each predictor variable at a time, keeping all the other predictor variables at their initial values. The x-axis represents the relative variable importance, the y-axis each variable - the wider the bar, the more impact the variable has on the model and the outcome. Consequently, the predictor variables are ranked from the most influential to the least influential.

## Discussion

The aim of the study was to identify the most important characteristics of offender patients with SSD who escaped or absconded from a forensic hospital by means of ML and to describe the best possible statistical model. The identified variables were *age at diagnosis of the SSD* and *age at admission*, *time spent in current hospitalization*, *problematic behavior during current hospitalization* (e.g., forbidden intake of drugs/ alcohol, breaking of rules, dissocial behavior), *amount of index offences, PANSS score at admission* and *discharge* (note: if the patient did not return after the event of escape or absconding, the last PANSS before the event was taken) and *dosing of neuroleptic medication* (daily cumulative olanzapine equivalent at discharge). The final model utilizing these variables achieved a balanced accuracy of 71% at an AUC of 0.75 with a sensitivity of 80% and a specificity of 60%.

The escaped or absconded offender patients suffering from SSD shared several characteristics with escapees or absconders identified in previous studies in mixed populations. Our results confirm studies showing that a younger age plays a central role in escape or absconding [1, 3–5, 9, 27, 29, 33–36]. Not only the age at the time of the incident was important as described in previous studies, but also a younger age at the time of the initial diagnosis of SSD. A shorter hospitalization period could also be confirmed [3, 4, 31, 33, 34, 38] indicating that escape and absconding arise in an earlier phase of treatment. Although, in contrast to previous investigations, no increased incidence of diagnoses of substance abuse was found [1, 4, 5, 23, 24, 33, 38–40], there was a higher incidence of substance abuse during the current forensic hospitalization. Interestingly, this behavior was embedded in a general behavior of dissociality and a proneness to rule breaking. In this context, the newly identified variable “number of index offences” may be a possible indicator for an increased propensity to breach rules even before treatment. However, a difference in the frequency of diagnosis of dissocial personality disorder between the groups was not apparent, since diagnosing a personality disorder in the presence of SSD is excluded according to ICD coding.

Although the evidence on the relationship between escape or absconding and symptoms of SSD is scarce [25], previous studies have reported that symptoms were exacerbated at the time of escape or absconding [19, 28, 31]. Based on the objective scores of the PANSS over the course of treatment, our findings indicate that patients who escaped or absconded not only had an acute exacerbation of symptoms at that time, but they generally displayed a more severe course of the disorder. Presumably, this is also reflected in a higher demand for neuroleptic drugs, which was found to be an influential factor in our sample. Our model did not identify any variables regarding childhood or adolescence and, with the exception of the two variables mentioned above, also no parameters from psychiatric or criminal history [1, 4, 5, 17, 19, 23, 24, 38]. Hence, our analysis indicates that past patient information is less relevant than information on the current hospitalization.

In summary, we found that offender patients with SSD who escape or abscond tend to be younger, have a more severe level of pathology and are more likely to exhibit dissocial behavior.

In general research on populations of offenders with SSD, especially in the context of violence, age [68–74], dissocial patterns [68–75], substance abuse [75–77] and psychotic symptoms [78–85] have also been identified as risk factors and dominate the field of forensic research on SSD and offending. Escape and absconding are rule-violating behaviors and thus perhaps a facet of dissocial behavior patterns that are interwoven with psychotic symptoms in a complex multifactorial framework that has not yet been sufficiently understood. It is possible that the group of offenders suffering from SSD comprises a subgroup of individuals who are particularly affected by these factors. For example, the framework by Hodgins’ [69] postulates a subgroup (“early starters”), which is characterized by young age, dissocial and rule breaking behavior and drug abuse. They may also be more prone to problematic behavior during hospitalization and more likely to escape or abscond, which should be investigated in future studies.

Although the goal of the study was not to develop a tool to identify patients who flee or escape, the usefulness of the current model and subsequent opportunities for the potential development of such a tool need to be discussed. This will be illustrated by a cost matrix applied to the results of the validation data set. Patients correctly identified as non-absconders/ non-escapees (TP - 60 patients) or absconders/ escapees (TN - 9 patients) have no direct negative impact on the hospital and therefore generate zero cost. Patients who are incorrectly identified as Absconder/Escapees (FN - 8 patients) may receive more extensive treatment and be unnecessarily restrained. This results in costs of 20 units per patient. Patients who are misclassified as non-absconders/ non-escapees and then nonetheless escape (FP - 6 patients) pose a massive risk to society and themselves [4, 23–25]. Here we postulate a cost of 100 units per patient. Calculating the total cost for our current model results in: 60 × 0 + 9 × 0 + 8 × 20 + 6 × 100 = 760 units. A perfect model with 100% specificity and sensitivity (0 FN/ 0 FP) results in no cost: 68 × 0 + 15 × 0 + 0 × 20 + 0 × 100 = 0 units. If the model correctly identified a patient who was misidentified as an absconder/escapee (sensitivity increase), this would result in the trade off of misclassifying a patient as a non-absconder/ non-escapee, resulting in an overlooked escape (specificity decrease) and in a cost of: 61 × 0 + 8 × 0 + 7 × 20 + 7 × 100 = 840 units. If we now turn these considerations around and increase specificity with a loss of sensitivity, i.e., identify absconder/escapees with more efficiency, this results in a cost of: 59 × 0 + 10 × 0 + 9 × 20 + 5 × 100 = 680 units. In summary, a decrease in specificity leads to an increase in undetected absconders/ escapees and a massive cost. In other words, for one missed absconder/ escapee, five patients may be erroneously treated too restrictively. Thus, these considerations show that detection of actual absconders and escapees are essential for model building. It also demonstrates that unbalanced data can lead to costly consequences. But it is not only these statistical considerations that must be considered when developing a potential tool. How expensive is it to treat a patient too restrictively, for example, and how much more expensive is it to have a patient drop out and potentially commit new crimes? At what level can we talk about acceptable risk and what is the minimum level of sensitivity and specificity that must be achieved? Rather complex questions that affect different areas like health and security policy, ethics must be addressed when developing and using a tool for risk assessment.

The results of the present explorative study may, nevertheless, provide benefits for clinical practice. By identifying a potentially problematic subgroup early in the course of hospitalization, the needs and risks of said subgroup can be addressed more effectively. A more individualized treatment of symptoms and an expansion of therapy to address dissocial behavioral patterns could possibly reduce the rate of escape and absconding.

However, since most of the risk assessment tools employed today serve as the basis for a variety of decisions about effective punishment and treatment [86], care must be taken to ensure that patients are not stigmatized or receive inferior treatment due to certain parameters like race or gender. Courts use such tools to assess the likelihood of recidivism or escape of pretrial detainees or offenders in bail and probation proceedings, or to set bail amounts [87]. ML methods tend to shift the trend of risk assessment toward prediction rather than intervention, as prediction-oriented risk assessments do not take into account the criminogenic nature of the criminal justice system itself, although the socially useful purpose of risk assessments should be directed more toward their utility as diagnostic and treatment guidance tools [86]. Bias can enter the design of a statistical model as well as the interpretation since the interpreter may more or less unconsciously incorporate personal political orientations, values and opinions when interpreting results [87]. In line with other authors [86–88], we emphasize the importance of providing decision makers, such as judges, with additional information about the limitations immanent in risk score predictions and encourage further efforts to better understand how risk assessment tools interact with biases and beliefs held by the individuals who rely on them.

In conclusion, although the performance parameters of our model seem to be reasonable, this sensitive topic with consequences for the medical treatment of humans is far from being an automated machine selection process. To achieve this, high sensitivity and specificity rates would be required. In this study, ML must be seen as an advanced statistical method for retrospective differentiation of individuals rather than a predictive modeling technique.

### Limitations

In addition to the well-known difficulties of retrospective data analysis (e.g., information loss), the database of 370 patients may be large for this specific group of offender patients with SSD, but is rather small for ML. This is accentuated by the fact that the group of patients who escaped or absconded was even smaller and artificially manipulated through imputation and balancing. Unbalanced data may cause algorithms to misrepresent the distributional properties of the data and lead to biased accuracies - this could be a problem in interpreting our final validation model. Previous research on the one hand recommends modeling with balanced data [89], and on the other hand advises against full artificial balancing as this can lead to overfitting [90]. Since we have chosen to balance the model as much as possible in the nested modeling, and at the same time significantly reducing the performance values of the model during validation, we have to consider overfitting in the modeling process. As this is the first ML model on this topic and a very specific population, further studies should test and validate this model on other (bigger and more balanced) patient populations, possibly employing a prospective study design and compare ML outcomes with clinical impressions of staff, to better assess true performance measures. This may help to identify other influencing factors and ultimately increase sensitivity and specificity. However, future studies may use the results of the present study, allowing more patients and fewer variables to be evaluated in order to improve the use of resources and build stronger models.

## Conclusion

For the first time it was possible to create and describe a model using innovative ML analysis to identify influential factors related to escape and absconding in forensic patients with SSD. Patients who escaped or absconded were generally younger and showed more pronounced pathological manifestations. They exhibited an overall more problematic and rule-breaking behavior. Early identification of these patients could help to prevent problematic events from occurring in the first place by providing specifically tailored treatment strategies for these patients.

## Supplementary Information

**Additional file 1.**

## Data Availability

The datasets generated and/or analysed during the current study are not publicly available due to the sensitive nature of the data concerning patients in a forensic hospital but are available from the corresponding author on reasonable request.

## Abbreviations

SSD: Schizophrenia spectrum disorder
PANSS: Positive and Negative Syndrome Scale
NHST: Null hypothesis significance testing
ICD: International Classification of Diseases
SD: Standard deviation
ML: Machine Learning
AUC: Area Under the Curve
PPV: Positive Predictive Value
NPV: Negative Predictive Value
