# Supplementary material for: Escape and absconding among offenders with schizophrenia spectrum disorder – an explorative analysis of characteristics
Source: BMC Psychiatry. 2021 Mar 4;21:122. doi: 10.1186/s12888-021-03117-1 (PMC7931588; doi:10.1186/s12888-021-03117-1)
Supplement: Supplementary file 1 — Additional file 1. [file 12888_2021_3117_MOESM1_ESM.docx]

**Escape and Absconding among offenders WITH SCHIZOPHRENIA spectrum disorder – an explorative analysis of characteristics**

Johannes Kirchebner, Steffen Lau, Martina Sonnweber

Supplementary Materials

Default hyperparameters for model building during nested cross validation on training dataset

| **Algorithm** | **Hyperparameter** |
| --- | --- |
| Logistic Regression | - |
| Tree | minisplit=20; cp=0.01; maxcomplete=4; maxsurrogate=5; usesurrogate=2; surrogatestyle=0; maxdepth=30;xval=10 |
| Random Forest | Ntree=500; replace=true; nodesize=1; importance=false; localImp=false; |
| Gradient Boosting | distribution=Bernoulli; n.tress=100; cvfolds=0; interaction.depth=1; n.minobsinnode=10; shrinkage=0.1; bag.fraction=0.5; train.fraction=1 |
| KNN | integer=7; numeric=2; logical=true |
| SVM | cost=1; nu=0.5; kernel=radial; degree=3; cachesize=40; tolerance=0.001; shrinking=true |
| Naive Bayes | laplace=0 |

Variance inflation factor (VIF) of multicollinearity test on validation dataset

| SD1 | 2.49 |
| --- | --- |
| PH1 | 2.56 |
| DZ7 | 1.46 |
| DZ10 | 1.64 |
| D1 | 1.13 |
| R9e | 1.09 |
| R20h | 1.10 |
| R22a | 1.09 |
| PA_A | 1.60 |
| PA_D | 1.60 |
